# Patents of Interest to Dentists Recently Granted

**Published:** 1907-12

**Authors:** 


### PATENTS.

PATENTS OF INTEREST TO DENTISTS RECENTLY GRANTED.

868964. Crown-pin for dental work, Henry D. Bultman, New York, N.Y.
869175. Toothpick, Peter Gorut, Fulton Chain. N. Y.
869191. Dental Plate, Gordon W. Morgan, Salem, Va.
869035. Dental Instrument, Wm. C. Wolford, Confluence, Pa.

869417. Stone Guard for dental grinding tools, Frank W. Chandler Valley City, N. D.

869840. Dental Engine, Winfield E. Hanson, Biddeford, Maine.

869563. Handpiece for Dental Engines, James F. Hardy, New York, N. Y.

869562. Dental Engine, James F. Hardy, New York, N. Y.

869573. Toothpick Machine, Wm. F. Hutchinson, Nyack, N. Y.

869520. Dental Impression Pliers, Myron A. Robbins, Passaic, N. Y.

869993. Machine for Boxing Toothpicks, Simon S. Tainter, W. W. Tainter, and G. P. Stanley, Dixfield, Maine.

870824. Handpiece for Dental Engines, James F. Hardy, New York, N. Y.

870825. Angle Attachment for Dental Handpieces, James F. Hardy, New York, N. Y.

870962. Manufacture of a new Material for use in Dentistry, Otto Hoffman, Charlottenburg, Germany.

870573. Instrument for Introducing Liquid Medicament into Teeth, Charles G. Myers, Cleveland, Ohio.

870909. Dental Articulator, George B. Snow, Buffalo, N. Y.

871400. Combination Dental Bracket, George Hall, Lima, Ohio.

871430. Artificial Tooth, Joseph Morris, North Wales, Pa.

Copies of above patents may be obtained for fifteen cents each, by addressing John A. Saul, Solictior of Patents, Fendall Building, Washington, D. C.

